# Treating critical supraventricular and ventricular arrhythmias

**DOI:** 10.4103/0974-2700.62114

**Published:** 2010

**Authors:** Hans-Joachim Trappe

**Affiliations:** Department of Cardiology and Angiology, University of Bochum, Germany

**Keywords:** AED, emergency medicine, intensive care, out-of-hospital cardiac arrest, tachyarrhythmias

## Abstract

Atrial fibrillation (AF), atrial flutter, AV-nodal reentry tachycardia with rapid ventricular response, atrial ectopic tachycardia and preexcitation syndromes combined with AF or ventricular tachyarrhythmias (VTA) are typical arrhythmias in intensive care patients (pts). Most frequently, the diagnosis of the underlying arrhythmia is possible from the physical examination (PE), the response to maneuvers or drugs and the 12-lead surface electrocardiogram. In unstable hemodynamics, immediate DC-cardioversion is indicated. Conversion of AF to sinus rhythm (SR) is possible using antiarrhythmic drugs. Amiodarone has a conversion rate in AF of up to 80%. Ibutilide represents a class III antiarrhythmic agent that has been reported to have conversion rates of 50-70%. Acute therapy of atrial flutter (Aflut) in intensive care pts depends on the clinical presentation. Atrial flutter can most often be successfully cardioverted to SR with DC-energies <50 joules. Ibutilide trials showed efficacy rates of 38-76% for conversion of Aflut to SR compared to conversion rates of 5-13% when intravenous flecainide, propafenone or verapamil was administered. In addition, high dose (2 mg) of ibutilide was more effective than sotalol (1.5 mg/kg) in conversion of Aflut to SR (70 versus 19%). Drugs like procainamide, sotalol, amiodarone or magnesium were recommended for treatment of VTA in intensive care pts. However, only amiodarone is today the drug of choice in VTA pts and also highly effective even in pts with defibrillation-resistant out-of-hospital cardiac arrest (CA). There is a general agreement that bystander first aid, defibrillation and advanced life support is essential for neurologic outcome in pts after cardiac arrest due to VTA. Public access defibrillation in the hands of trained laypersons seems to be an ideal approach in the treatment of ventricular fibrillation (VF). The use of automatic external defibrillators (AEDs) by basic life support ambulance providers or first responder (FR) in early defibrillation programs has been associated with a significant increase in survival rates (SRs). However, use of AEDs at home cannot be recommended.

## INTRODUCTION

Emergency medicine and critical care are fields that often require rapid diagnosis and intervention for specific situations.[1] These critical interventions can be life-saving or severely debilitating depending on their appropriateness and timeliness. In cardiac emergencies, accurate differentiation of ventricular and supraventricular tachyarrhythmias is essential for appropriate management.[1,2] Most frequently, the diagnosis of the underlying arrhythmia is readily apparent; however, occasionally it is necessary to use clues from the physical examination (PE), the response to maneuvers or drugs, in addition to the 12-lead surface electrocardiogram (ECG).[3,4] Treatment of cardiac arrhythmias in intensive care and emergency medicine is sometimes difficult. Correct therapy based on an understanding of the mechanism that caused the arrhythmia may not only be lifesaving in the immediate situation but may also improve the quality-of-life. The purpose of the present manuscript is to summarize new strategies for patients with supraventricular or ventricular tachyarrhythmias in intensive care or cardiac emergencies.

## INCIDENCE AND TYPE OF CARDIAC ARRHYTHMIAS IN CRITICALLY ILL PATIENTS

It is well known for many years that cardiac arrhythmias can occur in healthy people or in patients with cardiac or extracardiac diseases. Arrhythmias are well defined in patients after myocardial infarction, in patients with underlying cardiac or pulmonary disease and in patients after cardiac surgery or heart transplantation. However, only few data are available regarding incidence and type of arrhythmias in critically ill patients. Reinelt *et al*.[5] studied all consecutive arrhythmia episodes in critically ill patients in a medical-cardiology ICU between 1996 and 1999. All episodes of patients with new-onset of sustained arrhythmias (duration >30 s) were included which were either self-terminated or that required intervention. A total of 310 arrhythmia episodes were assessed during the study period in 133 patients with a mean of 2.91 episodes per patient (range 1-14). Admission diagnoses were cardiac (*n* = 48), cardiac surgery (*n* = 45), resuscitation (*n* = 12), pulmonary (*n* = 15), sepsis (*n* = 5), neurological (*n* = 2) and others (*n* = 6). Among the 310 arrhythmia episodes, there were 278 tachycardia (179 episodes regular, 97 episodes irregular) and 32 bradycardia events (heart rate <40 beats/min). There were 108 narrow-QRS complex tachycardia and 168 wide-QRS complex tachycardia with two episodes of primary ventricular fibrillation (VF). Among the 278 tachycardias, 135 episodes (48.6%) were ventricular, 13 episodes (4.7%) torsade de pointes tachycardia and 83 episodes (29.8%) atrial fibrillation, 10 episodes Aflut (3.6%), 21 supraventricular tachycardia (7.6%) and 2 ectopic atrial tachycardia (0.7%). Baine *et al*.[6] studied the incidence of arrhythmia types, hospital and intensive care stay in 144 512 elderly patients (age 65 years or older) with cardiac arrhythmias (time interval 1991-1998). In 1998, atrial fibrillation was the most frequent arrhythmia and accounted for 44.8% of the relevant discharges. Atrial flutter was observed in 5.2%, sinoatrial node dysfunction in 13.2% and complete AV-block in 5.8%. Supraventricular tachycardia (SVT) was observed in only 3.8%. Cardiac arrest was present in 1.3%, ventricular tachycardia (VT) in 6.9% and VF in 1.3%.

## ATRIAL FIBRILLATION

**Figure 1:**
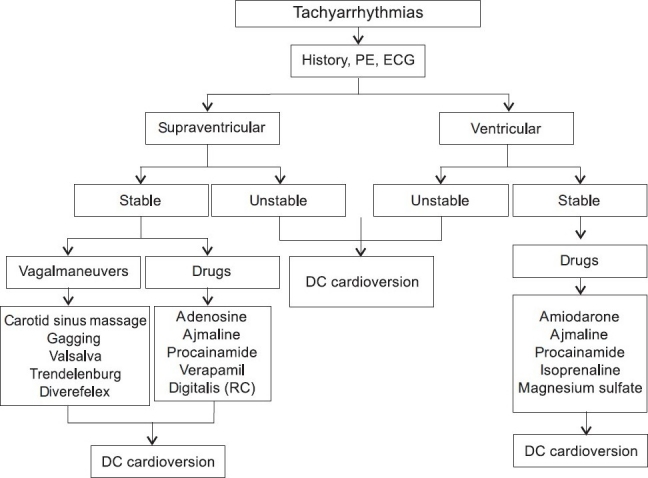
Treatment algorithm for patients with tachyarrhythmias. ACS = acute coronary syndrome, DC = direct current, ECG = electrocardiogram, PE = physical examination, RC = rate control

Atrial fibrillation is the most frequent arrhythmia in emergency rooms and intensive care units (ICUs), both in surgical and cardiology ICUs.[7] Knotzer *et al*.[8] found that 14.8% of ‘surgically ill patients’ developed atrial tachyarrhythmias compared to 47.4% of patients treated in a cardiology ICU. The goal of acute treatment of atrial fibrillation with rapid ventricular response is to restore sinus rhythm or to control the ventricular rate. If cardioversion to sinus rhythm is not possible, the secondary goal is to slow the ventricular response, usually to a rate of <100 beats per minute. Patients who are hemodynamically unstable (significant hypotension, severe angina, pulmonary edema) should be promptly cardioverted after administration of an anesthetic agent [Figure 1]. Cardioversion should always be performed in a synchronized mode.

### Restoration of sinus rhythm

The patients in whom atrial fibrillation is of known acute-onset (present for <48 h), DC-cardioversion should be considered early. Pharmacological conversion to sinus rhythm with antiarrhythmic drugs is a widely used therapeutic alternative with different efficacy rates.[9] Amiodarone has a conversion rate in atrial fibrillation of up to 80%.[10,11] In critically ill patients, amiodarone is indicated for a wide range of arrhythmias including recent- onset atrial fibrillation. Despite many publications on safety and efficacy of intravenous amiodarone in the treatment of patients with recent-onset atrial fibrillation, there is an increasing number of reports in critically ill patients highlighting occasional serious acute pulmonary toxicity.[12] Therefore, caution in the use of short-term administration of intravenous amiodarone in the critically ill patients with recent-onset atrial fibrillation is absolutely necessary and the duration of therapy should not exceed 24-48 h, except when absolutely necessary. In contrast to amiodarone, ibutilide represents another class III antiarrhythmic agent that has been reported to have high conversion rates.[13] Proarrhythmic effects occur in 5-8% of patients and careful monitoring is required. The conversion rates of recent-onset atrial fibrillation to sinus rhythm with ibutilide range from 50 to 70% and it seems that ibutilide is even successful when intravenous amiodarone fails to convert atrial fibrillation. Hennersdorf *et al*.[14] studied 26 patients in whom atrial fibrillation or flutter persisted for a maximum of 6 h. All patients initially received amiodarone (150 mg iv) and after 2 h of persistent arrhythmia ibutilide (1 mg iv; in the case of persisting arrhythmia and body weight >70 kg, a second infusion of 1 mg ibutilide was administered after 30 min). Before the administration of ibutilide, magnesium (1 g) was administered and high normal levels of potassium serum levels were achieved (4.5-5.0 mmol/l). After amiodarone administration, atrial fibrillation persisted in only 27% of patients. After administration of ibutilide, conversion to sinus rhythm was achieved in 82% patients after a median time of 7 min (range 3-12 min).

### Rate-control in atrial fibrillation with rapid ventricular response

A loss of atrial synchrony as well as the rapid ventricular response may be poorly tolerated in hemodynamically compromised patients. Attempts to restore sinus rhythm are frequently unsuccessful or even debatable.[15,16] Heart rate control (RC) becomes the main therapeutic goal in such situation. The optimal regimen for pharmacological RC during atrial fibrillation in critically ill patients is unclear. It is well known for many years that treatment with intravenous digoxin, verapamil, beta-blocking agents or diltiazem alone or in combination is effective in patients with atrial fibrillation and rapid ventricular response. Digoxin may be helpful for RC with an initial dose of 0.5 mg. After 30 min, 0.25 mg digoxin should be administered again. In intensive care and emergency medicine other than therapeutic strategies are verapamil (5-10 mg iv), diltiazem (20 mg iv) or beta-blocking agents as propranolol (1-5 mg iv, additional infusion of 10-120 mg per day) and esmolol (500 µg/kg over 1 min, followed by a 4-min maintenance infusion of 50 µg/kg/min with further dose adjustment as necessary). However, beta-blocking agents or calcium channel blockers may cause additional hypotension. Delle Karth *et al*.[17] compared another pharmacological approach for RC in critically ill patients; these authors studied the role of amiodarone or diltiazem in a prospective, randomized trial. Sixty patients with atrial tachyarrhythmias (atrial fibrillation 57 patients, Aflut 2 patients, atrial tachycardia 1 patient) were randomized to diltiazem (25 mg bolus followed by a continuous infusion of 20 mg/h for 24 h) (group I), amiodarone (300 mg bolus) (group II) or amiodarone (300 mg bolus followed by 45 mg/h for 24 h) (group III). The primary end point was a >30% heart rate reduction within 4 h. The secondary end point was a heart rate <120 beats/min. The primary end point was achieved in 70% of group I patients, 55% of patients in group II and in 75% of patients in group III (*P* = 0.38). In patients achieving heart RC, diltiazem showed a significantly better rate reduction when compared with group II and III (*P* < 0.01). However, premature drug discontinuation due to hypotension was required significantly more often in group I (30%) than in group II (0%) or group III (5%) (*P* < 0.01). The study showed that sufficient RC can be achieved in critically ill patients with atrial tachyarrhythmias using either diltiazem or amiodarone. Although diltiazem allowed significantly better 24-h heart RC, this effect was offset by a significantly higher incidence of hypotension requiring discontinuation of the drug.

## ATRIAL FLUTTER

Acute therapy for patients with Aflut in intensive care or emergencies depends on the clinical presentation. If the patient presents with acute hemodynamic collapse or congestive heart failure, emergent direct-current synchronized shock is indicated [Figure 1]. Atrial flutter can most often be successfully cardioverted to sinus rhythm with energies <50 J. A number of drugs have been shown to be effective in conversion of Aflut to sinus rhythm. Placebo-controlled intravenous ibutilide trials showed efficacy rates of 38-76% for conversion of Aflut to sinus rhythm.[18,19] For those who responded to ibutilide, the mean time interval to conversion was 30 min; the efficacy of intravenous ibutilide (76%) was significantly higher than that of intravenous procainamide (14%).[20] Several single-blinded, randomized control trials comparing intravenous flecainide with either intravenous propafenone or intravenous verapamil have shown relatively poor efficacy for acute conversion (5-13%); in addition, the conversion rate of intravenous sotalol varied from 20-40% depending on the sotalol dose, but was not different from placebo. High dose (2 mg) of ibutilide was more effective than sotalol (1.5 mg/kg) in conversion of patients with Aflut to sinus rhythm (70 versus 19%).[21]

## NARROW-QRS COMPLEX TACHYCARDIA

Narrow-QRS tachycardia is a cardiac rhythm with a rate faster than 100 beats per minute and a QRS duration of <0.12 s. The patient with narrow-QRS tachycardia usually seeks medical attention because of palpitations, light-headedness, shortness of breath or anxiety. In many patients with narrow-QRS complex tachycardia, the tachycardia rate is very high (180-240 beats per minute) and therefore, after onset of the tachycardia, the patient arrives very soon in an ICU for diagnosis and treatment.[1]

### Acute management

The definitive diagnosis can be made in >90% of the patients based on 12-lead ECG and clinical criteria. Acute treatment should be initiated based on the underlying mechanism. In regular narrow-QRS complex tachycardia, vagal maneuvers should be initiated to terminate the arrhythmia or to modify AV conduction [Figure 1]. If this fails, intravenous antiarrhythmic drugs should be administered for arrhythmia termination in hemodynamically stable patients. Adenosine, calcium channel blockers (verapamil) or beta-blocking agents are the drugs of first choice. The advantage of adenosine relative to intravenous calcium antagonists or beta-blockers relates to its rapidity of onset and short half-life.[3,4] Longer acting agents (intravenous calcium channel blockers or beta-blocking agents) are of value, particularly for patients with recurrences of narrow-QRS tachycardia. It is clear to avoid concomitant use of intravenous calcium channel blockers and beta-blocking agents because of possible increase of hypotensive and/or bradycardiac effects.[1]

## VENTRICULAR TACHYARRHYTHMIA

One of the most important problems in intensive care and emergencies are patients with recur rent VT, ventricular flutter or VF.[22] Management of cardiac arrest due to life-threatening ventricular tachyarrhythmias is a very important goal in these patients to avoid serious problems and to avoid sudden cardiac death.[23] However, treatment of the underlying arrhythmia required correct diagnosis in the majority of patients, possibly, using 12-lead surface ECG.[24] However, careful ECG interpretation of all 12 ECG leads is necessary for the correct diagnosis.

## WIDE-QRS COMPLEX TACHYCARDIA

Since a drug given for the treatment of SVT may be deleterious to a patient with VT, the differential diagnosis in broad QRS tachycardia is critical; wide-QRS complex tachycardias (QRS duration >0.12 s) often pose a difficult diagnostic and therapeutic problem.[25,26] Errors are made because emergency care professionals wrongly consider VT unlikely, if the patient is hemodynamically stable, and they are often unaware of the ECG findings that quickly and accurately distinguish VT in more than 90% of cases.[26] To make the right diagnosis, it is ideal to have a 12-lead-ECG. Diagnostic clues for differentiation of VT from SVT are findings in lead V1 and V6; in addition, a QRS of 0,14 sec or more favors a diagnosis of VT. There are several possible mechanisms of wide-QRS complex tachycardia [Table 1]. In intensive care and emergencies, it is necessary to divide wide-QRS complex tachycardia into those with monomorphic or polymorphic morphologies as well as torsade de pointes tachycardia. Different therapeutic strategies are necessary in these tachyarrhythmias.[27]

**Table 1:** Differential diagnosis of wide complex tachycardia

Ventricular tachycardia
Supraventricular tachycardia with aberrancy
Supraventricular tachycardia with preexisting bundle branch block
Antidromic supraventricular tachycardia using accessory pathways
Antegrade conduction over one bypass tract and retrograde conduction over another bypass tract
Atrial tachycardia with anterograde conduction over an accessory pathway
Atrioventricular nodal reentry with anterograde conduction down a bystander bypass tract

### Acute management

The initial approach depends on the hemodynamic severity and symptoms associated with the tachycardia. When the patient is hemodynamically unstable or is in pulmonary edema, the tachycardia should be promptly cardioverted with a direct-current synchronized shock [Figure 1]. Once the hemodynamically stable patient has been cardioverted and stabilized, it is important to evaluate the pre-conversion 12-lead-ECG for QRS configuration and signs of AV dissociation.

### Wide-QRS tachycardia with hemodynamically stable situation

For hemodynamically stable patients, a 12-lead-ECG should allow an accurate diagnosis in the majority of patients. If after analysing the ECG the diagnosis is uncertain, the patient should be treated for VT. This is by far the most common diagnosis in patients with wide complex tachycardia and VT is potentially life-threatening.[28,29]

### Monomorphic ventricular tachycardia

**Figure 2:**
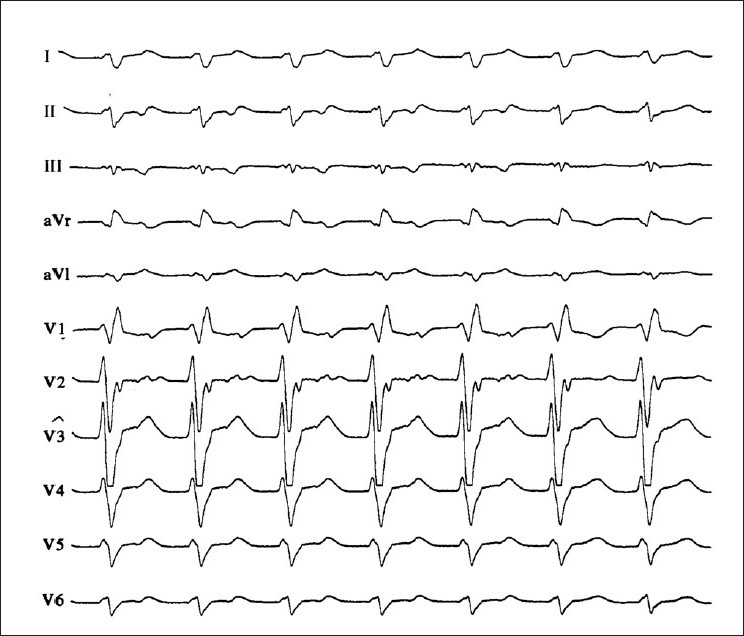
12-lead-ECG of a patient with wide QRS complex tachycardia. The tracing shows right bundle branch block morphology. Note the typical QRS features in leads V1 and V6 (triphasic appearance of V1, R to S ratio of less than 1 in V6). The AV dissociation and the northwest axis are also helpful clues for the diagnosis of ventricular tachycardia

In patients with sustained (duration >30 s) hemodynamically stable monomorphic VT, amiodarone plays an important role to terminate VT (150-300 mg in 5 min iv, followed by an infusion of 1050 mg/day). Alternatives is the administration of procainamide (10 mg/kg iv) or ajmaline (50-100 mg iv over 5 min) with high termination rates [Figure 2]. In patients with VT and in the setting of acute myocardial ischemia, lidocaine (100-150 mg iv) was the treatment of choice for long term. However, it is well known that the efficacy of ajmaline is higher than the effect of lidocaine, whereas lidocaine is associated with high risk for degeneration of monomorphic VT into VF.[25] Therefore, lidocaine is no more indicated in these patients and should be avoided. Other antiarrhythmic drugs like sotalol (20 mg in 5 min iv), propafenone (1-2 mg/kg iv) or flecainide (1-2 mg/kg iv) do not play a role as ‘first line’ drugs in stable monomorphic VT.[30–32]

### Polymorphic ventricular tachycardia

**Figure 3:**
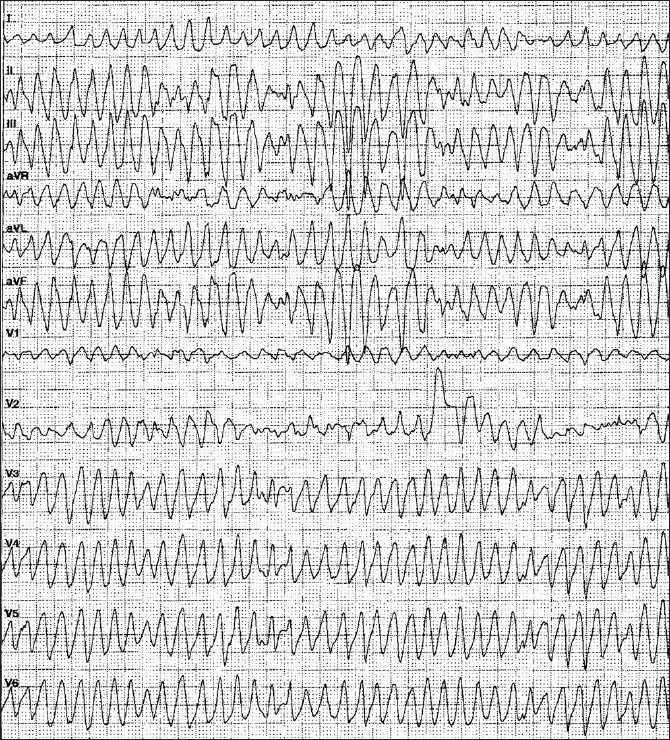
12-lead-ECG of a patient with fast polymorphic ventricular tachycardia after acute myocardial infarction.

Less frequently, patients may present with polymorphic VT [Figure 3]. Factors associated with this arrhythmia are electrolyte abnormalities, acute myocardial ischemia, reperfusion arrhythmia, proarrhythmia from type Ic antiarrhythmic drugs or QT prolongation.[28,29] The treatment of choice of polymorphic VT is, after coronary care unit monitoring, discontinuation of the offending drug and isoproterenol infusion with shortening repolarization and increasing the heart rate (1-4 µg/min iv) as initial steps. Alternative treatment with similar effects is administration of atropine (0.5-1.0 mg iv, maximum 0.04 mg/ kg iv). Atrial or ventricular pacing will often suppress the polymorphic VT. In these situations with polymorphic VT, there is also a clear indication for emergency treatment with amiodarone (150-300 mg iv, followed by infusion of 1020 mg/ day).[27,31] Despite all considerations about the ‘ideal’ therapeutic strategy, in polymorphic VT patients, evaluation of the underlying disease and the mechanism of the arrhythmia is the most important step. In some cases, acute myocardial ischemia is present (‘acute coronary syndrome’, ACS) and reperfusion therapy (PCI, thrombolysis, bypass grafting) will succeed and terminate the arrhythmia.[33] Of course, in these cases reperfusion therapy is indicated and no antiarrhythmic drug treatment is necessary.

### Torsade de pointes tachycardia

Torsade de pointes is a type of polymorphic VT associated with marked QT prolongation. It may occur after administration of class Ia and class III antiarrhythmic drugs.[28,30] The tachycardia is paroxysmal and may result in VF and sudden death.[34,35] Its onset is promoted by a slow basic rhythm and frequently follows a pause induced by a premature ventricular contraction. The tachycardia is characterized by polymorphic QRS complexes. Progressive lengthening of the QT interval and the development of a prominent U wave are important warning signs. The degree of QT prolongation that predicts torsade de pointes tachycardia is not known. However, prolonged QT syndromes may be congenital (Romano-Ward syndrome, Jervell-Lange-Nielsen syndrome) or acquired (class I and class III antiarrhythmic drugs). The treatment in intensive care and emergencies is discontinuation of the offending drug and correction of electrolyte abnormalities with potassium and magnesium.[27] Intravenous magnesium sulfate (initial bolus of 2 g iv, another bolus of 2 g after 15 min if the initial bolus failed, followed by a continuous infusion of 500 mg/h iv) may be efficacious, even if the serum magnesium level is within the normal range.[36,37] Although magnesium has been used to treat arrhythmias for several decades, its mechanism of action and efficacy remain controversial.[38,39] In addition, the recommended use of magnesium has not undergone scrutiny by randomized trials.[40]

## VENTRICULAR FIBRILLATION AND CARDIAC ARREST

Approximately 1,000 people in the United States suffer from cardiac arrest each day, most often as a complication of an acute myocardial infarction with accompanying VF or uns VT. In 2005, the American Heart Association (AHA) reported again the chain of survival concept, with four links-early access, cardiopulmonary resuscitation, defibrillation and advanced care – as the way to approach cardiac arrest.[41] It has been pointed out that the highest potential survival rate (SR) from cardiac arrest can be achieved only when the following sequence of events occurs as rapidly as possible: (a) recognition of early warning signs, (b) activation of the emergency medical services system, (c) basic cardiopulmonary resuscitation, (d) defibrillation, (e) management of the airway and ventilation and (f) intravenous administration of medications.

### Neurologic outcome

There is general agreement that bystander first aid, defibrillation and advanced life support is essential for neurologic outcome in patients after cardiac arrest. Bur *et al*.[42] evaluated the effects of basic life support (BLS), time to first defibrillation and emergency medical service arrival on neurologic outcome in 276 patients after cardiac arrest. In contrast to intubation (odds ratio 1.08; 95% CI, 0.51-2.31; *P* = 0.84), basic life support (odds ratio 0.44; 95% CI, 0.24-0.77; *P* = 0.004) and time to first defibrillation (odds ratio 1.08; 95% CI, 1.03-1.13; *P* = 0.001) were significantly correlated with good neurologic outcome. In addition to the better neurologic outcome, among the patients who did not receive BLS the average cost per patient with good neurologic outcome significantly increased with the delay of the first defibrillation *(P* < 0.001). The importance of cerebral perfusion and pressure and cerebral tissue oxygen tension during cardiopulmonary resuscitation has been described elsewhere.[43]

## EARLY DEFIBRILLATION

Public access defibrillation, which places automatic external defibrillators (AED) in the hands of trained laypersons has the potential to be the single greatest advance in the treatment of VF since the development of cardiopulmonary resuscitation. Time to defibrillation is the most important determinant of survival from cardiac arrest.[44] The earlier the defibrillation is performed the better the success rates for resuscitation, irrespective of who is doing the first defibrillation.[45] In the last few years, there has been a significant increase in the use of AEDs in early defibrillation programs in a variety of settings, including hospitals, emergency medical service, police departments, casinos, airport terminals and commercial aircraft, among others. In most of these settings, use of AEDs by basic life support ambulance providers or first responder (FR) in early defibrillation programs has been associated with a significant increase in SRs.[46–48]

### World wide experience

**Figure 4:**
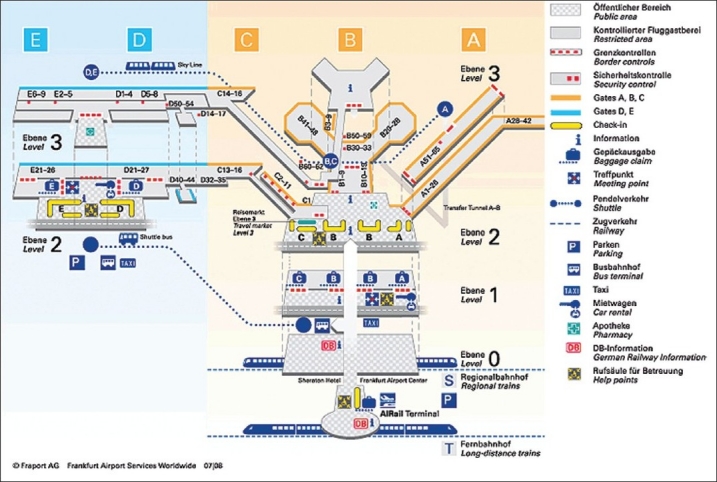
Map of the Rhein-Main Airport Frankfurt/Main showing the locations of automated external defibrillators in the terminals 1 and 2

**Figure 5:**
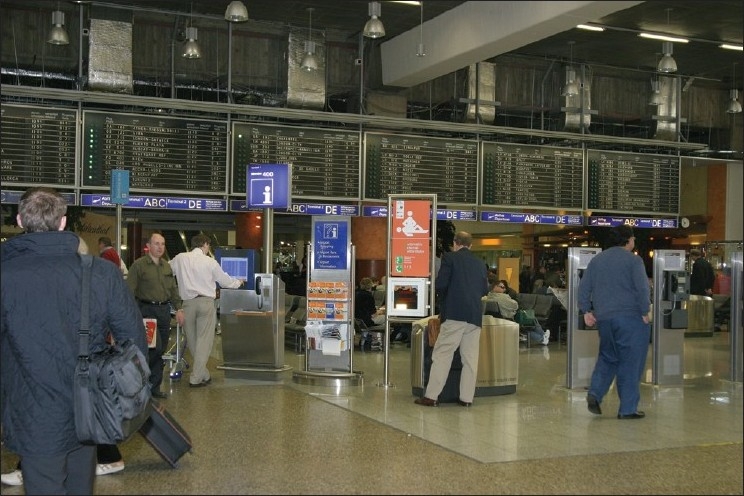
Example of an automated external defibrillator in the hall B (international flights) of the Rhein-Main Airport Frankfurt/Main

**Figure 6:**
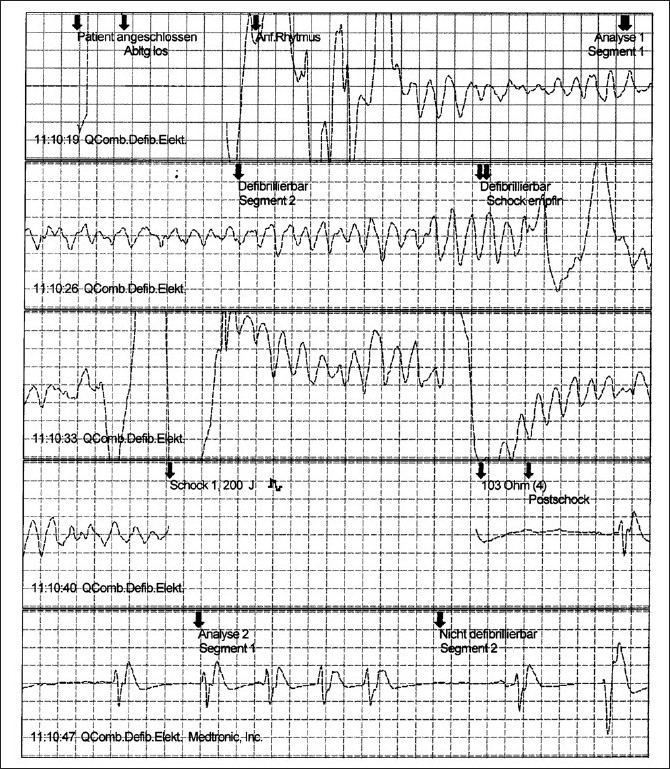
Ventricular fibrillation recorded in the terminal 1 of the Rhein-Main Airport. Ventricular fibrillation was successfully carried out in this passenger after a total recording of 21 s, resulting in a pause followed by sinus rhythmus. This passenger survived to be discharged from the hospital. The labels shown here depict the activity of the device as displayed on the electrocardiographic tracing

**Figure 7:**
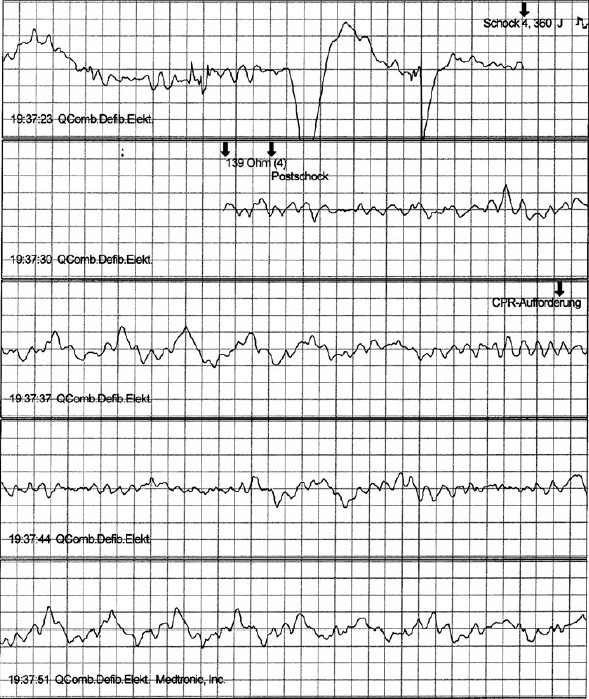
Ventricular fibrillation recorded in the terminal 2 of the Rhein-Main Airport in a passenger with known coronary artery disease and old myocardial infarction. Ventricular fibrillation was not converted in this passenger and after four shocks cardiopulmonary resuscitation was advised by the AED. Despite cardiopulmonary resuscitation by first responders and professionals, the passenger died due to electromechanical dissociation

Automatic external defibrillators (AEDs) were used in 105 patients with VF suffered in casinos.[46] Fifty-six of the patients (53%) survived to discharge from the hospital. Among the 90 patients whose collapse was witnessed (86%), the clinically relevant time intervals were a mean of 3.5 + 2.9 min from collapse to the delivery of the first defibrillation shock, and 9.8 + 4.3 min from collapse to the arrival of the paramedics (PMs). The SR was 74% for those who received his/her first defibrillation no later than three minutes after a witnessed collapse and 49% for those who received his/her first defibrillation after more than three minutes. Caffrey *et al*. reported the public use of AEDs in three Chicago airports.[48] During a two-year period, 21 persons had nontraumatic cardiac arrest, 18 of whom had VF. In the case of four patients with VF, defibrillators were neither nearby nor used within five minutes, and none of these patients survived. Three others remained in VF and eventually died later, despite the rapid use of a defibrillator within five minutes. Eleven patients with VF were successfully resuscitated, including eight who regained consciousness before hospital admission. No shock was delivered in four cases of suspected cardiac arrest, and the device correctly indicated that the problem was not due to VF [Tables 2 and 3].[49–57] In Germany, two AED projects were performed in Frankfurt airport (Fraport) with 50 million passengers per year and in LAGO-Die Therme, a well known European water park with approximately 700 000 visitors per year.[58,59] At Fraport, 16 AEDs were placed in the terminals 1 and 2 and 2000 FRs were trained in AED use and cardiopulmonary resuscitation [Figures 4 and 5]. During the 12-month-period, 5 passengers had out-of-hospital cardiac arrest (CA) after arrival of the airplane in terminals 1 or 2. Four patients were successfully resuscitated by FRs and AEDs (80%), while a 63-year-old men with coronary artery disease died from electromechanical dissociation despite cardiopulmonary resuscitation by FRs and emergency medical system [Figures 6 and 7]. In the LAGO project with 205 visitors in three years nobody died. Automatic external defibrillators were used in two visitors with non-arrhythmogenic syncope; no AED shock was delivered. The locations where the defibrillators were stored were chosen to make possible a target interval of 60 s from collapse to first defibrillation. Twenty water park officers were instructed in cardiopulmonary resuscitation and in the use of the AED.

**Table 2:** AED programs in communities

Author	Study-design	Patients	SR%	*P*
Eisenberg [49]	Paramedics + BLS	179 mit CA	18	< 0.05
	vs			
	BLS + AED		38	
Kellermann [50]	Paramedics	610337 IN	10	*
	vs			
	FR + AED		14	
Myerburg [51]	Police + AED	1181612 IN	17	0.047
	vs			
	Paramedics-AED		9	
Capucci [52]	FR + AED	173114 IN	11	0.006
	vs			
	PM		3	
Weaver [53]	FM + BLS	1287 IN	19	*
	vs			
	FM + AED		30	
Mosesso [54]	Police + AED	7 Städtische Gemeinden	26	0.01
	vs			
	PM		3	
PAD Trial [55^]^	BLS	993 Gemeinden	14	0.03
	vs	in 24 Regionen		
	BLS + AED	Nordamerikas	23	
Van Alem [56]	FM + police	469 CA	25	NS
	vs			
	PM		21	

AED = AUTOMATED EXTERNAL DEFIBRILLATOR, BLS = BASIC LIFE SUPPORT, CA = OUT-OF-HOSPITAL CARDIAC ARREST, FR = FIRST RESPONDER, IN = INHIBITANTS, FM = FIREMAN, PM = PARAMEDICS, NS = NON SIGNIFICANT SR = SURVIVAL RATE

* = NO DATA

**Table 3:** AED programs in public places

Author	No. of persons AED-location	AED-user	Patients	SR%
Venezuela [46]	Casinos	FR	105 pts VF	53
Caffrey [48]	100 mill pass, AI O'Hare, Midway, Meig Field (per year)	FR/ Paramedics	18 pts CA	56
Page [47]	70801874 pass 727956 flights American Airlines (per year)	FR	14 pts VF	40
O'Rourke [57]	31 mill pass 203191 flights Quantas (per year)	FR	46 pts CA	26
Trappe [58]	50 mill pass Frankfurt airport (per year)	FR	5 pts CA	80
Trappe [59]	2,05 mill visitors LAGO (three years)	FR	0 pt	*

AED = AUTOMATED EXTERNAL DEFIBRILLATOR, FR = FIRST RESPONDER, AI = AIRPORT, CA = OUT-OF-HOSPITAL CARDIAC ARREST, VF = VENTRICULAR FIBRILLATION, MILL = MILLIONS, pPASS = PASSANGERS, SR = SURVIVAL RATE

## WHAT ANTIARRHYTHMIC DRUGS DO WE HAVE IN INTENSIVE CARE AND EMERGENCIES?

Prompt cardiopulmonary resuscitation and early defibrillation either by DC-countershock or an AED significantly improve the likelihood of successful resuscitation from VF. Despite these circumstances, there is still a place for antiarrhythmic drugs to treat patients with life-threatening ventricular tachyarrhythmias like monomorphic or polymorphic VT in intensive care or emergencies.[4,41]

### Class I antiarrhythmic drugs

Use of *procainamide* is indicated in patients with VT. Its utility in pulseless cardiac arrest is less well studied and limited by the need for a relatively slow infusion (maximum rate 50 mg/ min) and the achievement of potentially toxic concentrations if administered more rapidly. Procainamide should be avoided in patients with preexisting QT prolongation and torsade de pointes tachycardia. The ECG and blood pressure must be monitored continuously during procainamide administration. Precipitous hypotension may occur if the drug is injected too rapidly[41] *Propafenone* and *flecainide* are class I antiarrhythmic dr ugs with significant conduction-slowing and neg ative inotropic effects. In addition, propafenone has nonselective beta-blocking properties. Intravenous propafenone is used for the same indication as flecainide and is acceptable for treatment of both SVT and VT. Because of significant negative inotropic effects, propafenone and flecainide should not be given to patients with impaired left ventricular function. In addition, both propafenone and flecainide should be avoided when coronary artery disease is suspected.

### Class III antiarrhythmic drugs

*Sotalol* prolongs action potential duration and increases cardiac tissue refractoriness. In addition, it has nonselective beta-blocking properties. Sotalol is effective in both SVT and VT. Side effects include bradycardia, hypotension and torsade de pointes tachycardia, Intravenous sotalol in intensive care medicine is limited by its need to be infused relatively slowly. This may be impractical and has uncertain efficacy in emergent circumstances, particularly under compromised circulatory conditions.[41] *Amiodarone* is a complex drug with effects on sodium, potassium and calcium channels as well as alpha- and beta-adrenergic blocking proper ties. Amiodarone is a highly efficacious antiarrhythmic agent for many cardiac arrhythmias, ranging from atrial fibrillation to malignant ventricular tachyarrhythmias.[60] In most published studies, intravenous amiodarone has been administered in patients with ventricular tachyarrhythmias only after failure of other antiarrhythmic drugs. In 1999, Kudenchuk described in 504 randomized patients with out-of-hospital CA due to refractory ventricular arrhythmias (ARREST study) that treatment with amiodarone (single 300 mg dose of intravenous amiodarone) resulted in a higher rate of survival to hospital admission (44%) compared to placebo (34%) *(P* = 0.03).[61] The role of amiodarone as an emergency drug has been reported recently by Taylor.[60] Today, amiodarone is the drug of choice for patients with VT and patients with VF when defibrillation failed.

### Magnesium

Severe magnesium deficiency is associated with cardiac arrhythmias, symptoms of heart failure and sudden cardiac death. Hypomagnesaemia can precipitate refractory VF and can hinder the replenishment of intracellular potassium. Magnesium deficiency should be corrected in intensive care and emergencies if present. Magnesium is not usually categorized as an antiarrhythmic agent. However, it is known for long time that in emergent circumstances, magnesium sulfate 1 to 2 g is helpful to suppress life-threatening ventricular tachyarrhythmias and should be administered over 1 to 2 min.[4] The role of magnesium in intensive care and emergency medicine has been described by Kaye and O'Sullivan in 2002.[42] These authors concluded that magnesium should be the first line therapy in eclampsia and torsade de pointes tachycardia. Data from other studies show that magnesium has a clearly defined role as a second line therapy in acute severe bronchial asthma.[40] Hypomagnesemia should be considered in patients with biventricular failure presenting with malignant ventricular tachyarrhythmias. It has been pointed out, that magnesium is safe and easy to use and should be available for immediate use in all ICUs and emergency departments.[62]

## CONCLUSIONS

Emergency medicine and critical care are fields that often require rapid diagnosis and intervention for specific situations. It is well known that in all patients with tachyarrhythmias, evaluation of the underlying etiology and the degree of left ventricular function (dysfunction) is essential. Correct treatment of arrhythmias in the intensive care patient is based on an understanding of the mechanism that caused the situation. The therapeutic role of antiarrhythmic drugs in the management of atrial fibrillation or cardiac arrest is debatable. The restoration of sinus rhythm is not the goal in any patient with atrial fibrillation and rhythm control is an alternative way for these patients. In victims of cardiac arrest, the prophylactic administration of antiarrhythmic drugs has never been studied formally. The optimal dose of antiarrhythmic drugs, whether best given before or only after multiple defibrillation shocks have failed to restore circulation, is not yet known. One of the most important points is to reduce the incidence of sudden cardiac death, both outside and in hospitals by the use of automated external defibrillators.[63–65] However, it is now well known that this therapy is not able to prevent sudden death when AEDs are installed at home and used by relatives.[66] Other strategies for cardiopulmonary resuscitation are essential to improve survival.[67,68]
